# Future directions and challenges in sports cardiology: interplay between blood pressure response and aortic remodeling in athletes

**DOI:** 10.1007/s00392-025-02653-0

**Published:** 2025-04-17

**Authors:** Pascal Bauer, Astrid Most

**Affiliations:** Department of Cardiology and Angiology, Justus- Liebig- University Giessen, 35390 Giessen, Germany

**Keywords:** SBP/MET slope, Elite athletes, Aortic root diameter, Exaggerated blood pressure, Vascular function, Central blood pressure, Genetic polymorphisms, Epigenetic effects of exercise

Sirs,

We sincerely appreciate the thoughtful comments raised by the correspondents [1] regarding our paper "Association of aortic root diameter and vascular function with an exaggerated blood pressure response to exercise among elite athletes" [2]. Their remarks highlight valuable considerations concerning genetic predispositions, the diversity of sports disciplines studied, and the importance of longitudinal data.

First, the correspondents correctly pointed out that our study did not consider genetic polymorphisms that may potentially influence vascular remodeling or blood pressure responses. Indeed, genetic polymorphisms play a substantial role in determining variability in cardiovascular adaptations to exercise and in modulating the blood pressure response during exercise [3–5]. Concurrently, epigenetic mechanisms mediate the long-term effects of physical activity on cardiovascular health by regulating genes involved in inflammation, metabolism, and cardiac function [6]. These exercise-induced epigenetic modifications not only help to explain inter-individual variability in training responses but also contribute to the protective effects of regular physical activity against cardiovascular disease. Hence, incorporating both genetic and epigenetic insights in future investigations could enhance our ability to predict individual cardiovascular adaptations and guide personalized exercise prescriptions [7].

However, routine genetic analyses are currently not integrated into standard pre-participation examinations in sports medicine practice, primarily due to logistical, ethical, and cost-related constraints. Consequently, physicians performing pre-participation screenings require data that accurately reflect clinical realities without reliance on specialized genetic tests. Moreover, even when genetic predispositions are known, recent research underscores that the type and intensity of sport participation critically modulate cardiovascular adaptations [8, 9]. Therefore, phenotype-based assessments remain indispensable in clinical decision-making, independent of genetic screening.

Second, the correspondents noted our exclusive focus on athletes involved in team sports, thereby excluding endurance disciplines such as cycling or marathon running. We fully agree that cardiovascular remodeling varies significantly across different sports due to differences in training volume, intensity, and hemodynamic load [8–10]. Our study specifically targeted mixed team sports to control for heterogeneity in training stimuli. Future research would undoubtedly benefit from broader sport-specific comparisons, thereby extending the applicability of our findings across a wider spectrum of athlete populations.

Third, our cross-sectional study design inherently limits our ability to draw conclusions regarding the long-term effects of an exaggerated blood pressure response (eBPR) on aortic remodeling and cardiovascular outcomes post-career. We acknowledge that longitudinal research is essential for evaluating the progression and clinical significance of aortic dilation. Therefore, our study group is currently collecting respective data of elite athletes. Recent longitudinal studies provide preliminary reassurance, suggesting that mild aortic dilations in young athletes may remain stable over medium-term follow-ups [11], although data from older endurance athletes indicate a potential for progressive aortic enlargement following prolonged exposure to intensive training [12]. Thus, we fully concur that prospective, long-term follow-up studies are required to clarify whether eBPR predicts future pathological remodeling or adverse clinical outcomes.

In conclusion, we encourage future research to integrate genetic and epigenetic assessments, include a broader range of sports disciplines, and employ longitudinal evaluations to further elucidate these associations.

## Data Availability

The manuscript data will not be deposited.
